# P-300. Antimicrobial Resistance Focus in French ICU: A national surveillance network analysis on *Pseudomonas aeruginosa* nosocomial infections

**DOI:** 10.1093/ofid/ofae631.503

**Published:** 2025-01-29

**Authors:** Xavier Bourge, Arnaud Friggeri, Alain Lepape, Claire Prevot, Anais Machut, Charles-Hervé Vacheron

**Affiliations:** MSD France, VOURLES, Rhone-Alpes, France; Hospices Civils de Lyon, LYON, Rhone-Alpes, France; Centre Hospitalier Lyon Sud, Hospices Civils de Lyon, Lyon, Rhone-Alpes, France; MSD France, VOURLES, Rhone-Alpes, France; Hospices Civils de Lyon, LYON, Rhone-Alpes, France; Hospices Civils de Lyon, LYON, Rhone-Alpes, France

## Abstract

**Background:**

The aim of this study was to perform a comprehensive description on a cohort of *Pseudomonas aeruginosa* infected patients in ICU, comparing their characteristics between susceptible and resistant strains, to understand the impact of resistance on clinical decisions and to characterize patients at risk of MDR.

Site of infection of Pseudomonas aeruginosa

**Figure:**
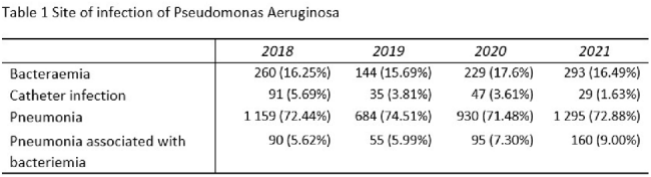

**Methods:**

Adult patients hospitalized in France for at least 48 hours in ICU participating to REA-REZO surveillance between 2018 and 2021 were included in the data base used to create this cohort.

Resistance patterns of Pseudomonas aeruginosa

**Figure:**
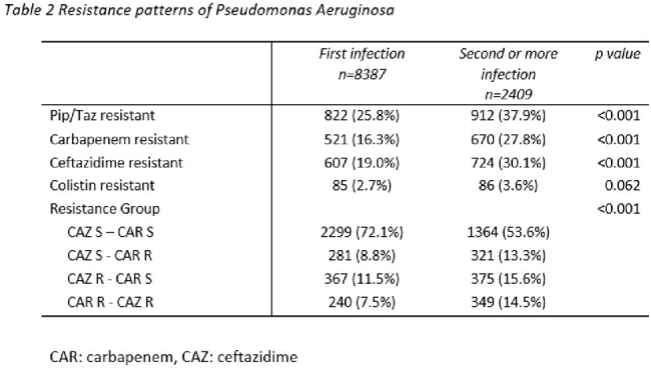

**Results:**

4196 patients with nosocomial *Pseudomonas aeruginosa* infections were included in this cohort. Most infections were identified in patients with pneumonia, with a consistently high prevalence ranging from 71.5% to 74.5% depending on the year.

Among the Pseudomonas, 1734 (31.0%) were resistant to piperacillin/tazobactam, 1331 (23.8%) were resistant to ceftazidime, and 1191 (21.3%) were carbapenem resistant. A statistically significant difference (p< 0.001) in resistance patterns between the first and subsequent infections is seen, notably for resistance to carbapenems which rose markedly from initial to subsequent infections, from 16.3% to 27.8% (p< 0.001).

Regarding occurrence of resistant Pseudomonas strains, statistically significant differences in age distribution among the resistance groups are noted (p< 0.001), particularly evident in patients under 55, where 24.0% of those with dual resistance are under this age threshold, compared to 19.7% with no resistance. The SAPS II score was higher among resistant strains, as well as the duration in ICU stay, suggesting more challenging infections.

Description of the patient with Pseudomonas aeruginosa infections

**Figure:**
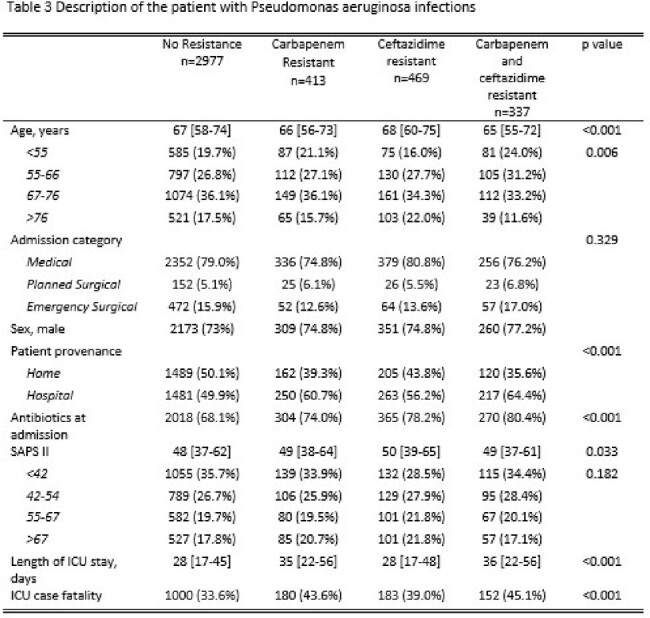

**Conclusion:**

These results reaffirm the critical challenge posed by resistant Pseudomonas in the ICU. It highlights the need for comprehensive antibiotic strategies that address the clinical, psychological, and economic dimensions of this issue.

**Disclosures:**

**Xavier Bourge, PharmD**, MSD France: Employee **Arnaud Friggeri, MD**, MSD France: Advisor/Consultant **Alain Lepape, MD**, MSD France: Grant/Research Support **Claire Prevot, n/a**, MSD France: Employee **Anais Machut, n/a**, MSD France: Grant/Research Support **Charles-Hervé Vacheron, MD**, MSD France: Grant/Research Support

